# Frontal skull craniotomy combined with moderate-dose radiotherapy effectively ameliorate a rare case of non-secretory, multiple myeloma with orbital involvement

**DOI:** 10.1186/1477-7819-7-86

**Published:** 2009-11-12

**Authors:** Hui-Ling Ko, Ching-Lin Chen, Kwan-Hwa Chi

**Affiliations:** 1 Departments of Radiation Therapy and Oncology, Taipei, Taiwan; 2 Department of Neurosurgery, Shin Kong Memorial Hospital, Taipei, Taiwan

## Abstract

**Background:**

Orbital infiltration in patients with multiple myeloma is a rare condition, with less than 50 cases reported in the medical literature. Most patients undergo conservative treatment because multiple myeloma is a disseminated systemic disease.

**Case presentation:**

A 43-year-old male subject with multiple myeloma and long-term survival presented with orbital involvement. The subject lacked the typical features and poor prognostic factors associated with multiple myeloma, such as renal failure, hypercalcemia, and paraprotein in the serum and urine. The orbital computed tomographic scan revealed the tumor encasing the optic nerve, but without prominent bony destruction. Therefore, a frontal skull craniotomy with an epidural entrance to the orbital space was performed, to completely extirpate the orbital mass. The surgical procedure was followed by moderate-dose radiation therapy. After 32 months of follow-up care, the subject is doing well with excellent local control.

**Conclusion:**

Although the effectiveness and applicability of this approach remains to be determined, this case report demonstrates that accurate and early detection combined with local surgical treatment and appropriate radio/chemotherapy, can be applied to effectively extend an orbital multiple myeloma patient's life.

## Background

Multiple myeloma presents as a systemic, disseminated disease and represents an uncontrolled proliferation of plasma cells with the overproduction of proteins belonging to the immunoglobulin family, and accounts for 1% to 2% of all cancers [1]. The median age at diagnosis is the sixth decade, and there is a progressive increase in incidence with age, reaching a maximum in the seventh decade of life. Multiple myeloma is characterized by the neoplastic proliferation of a single clone of plasma cells, leading to enhanced levels of paraprotein in serum and/or urine. The plasma cells proliferate in the bone marrow and frequently invade adjacent bone, causing skeletal destruction that results in bone pain and pathological fractures. Bone marrow involvement may be focal rather than diffuse, which requires repeated bone marrow examinations in order to obtain an accurate diagnosis [1].

A report from the International Myeloma Working Group in 2003 [2], indicates that urine contains paraprotein in approximately 75% of patients. Ninety-seven percent of patients with multiple myeloma have paraprotein in the serum or urine at the time of diagnosis. However, a minority of patients with non-secretory myeloma have no monoclonal protein. Only 3% of patients with symptomatic multiple myeloma are found to have no paraprotein in their plasma cells [3]. Treatment for non-secretory myeloma is similar to that for multiple myeloma, and both share a similar response to therapy and survival rate.

Plasma cell myeloma, or orbital myelomatosis is a rare and diverse condition, even among patients with known multiple myeloma [4]. The differential diagnosis of orbital tumors includes metastatic carcinoma, malignant lymphoma, optic pseudotumor, lacrimal gland tumors, as well as plasma cell tumors. It is estimated that fewer than 50 cases of orbital myelomatosis have been reported in the medical literature [5]. A plasmacytoma of the orbit presents with non-specific symptoms, including proptosis, a change in visual acuity, displacement of the orbit, and diplopia. In fact, 80% of patients with orbital multiple myeloma present with proptosis, although visual impairment, varying from minor defects to complete blindness, are also often observed [5]. The tumors typically arise from the bone and extend into the soft tissue. Accurate diagnosis of an orbital tumor arising from myeloma is particularly challenging in patients who do not have a history of multiple myeloma.

In the current report, we present a patient with multiple myeloma who had an unusual presentation, and has experienced an extended survival after undergoing a frontal skull craniotomy followed by moderate-dose radiotherapy.

## Case presentation

The subject examined herein was a 43-year-old male factory worker. Seven years before this study was initiated, he had a motorcycle accident and experienced pain in the left hip for which he was referred to our Emergency Department (ED). A left hip intertrochanteric fracture was suspected based on radiologic observations. A curettage with internal fixation of the left femur was performed, and the pathologic report noted lobules of mature plasma cells in the bone and soft tissue. Further assessment of the condition was carried out post-surgery. A standard skull X-ray series revealed typical "punched out" osteolytic lesions. Systemic disease was considered, however, bone marrow analysis was normal and no abnormal monoclonal gammopathy was detected by serum immunoelectrophoresis. Blood analysis, including calcium level and complete blood count were normal. In addition, no serum paraprotein or urinary free light chains were detected. Therefore, non-secretory multiple myeloma was proposed and the patient received radiotherapy (5040 cGy in 28 fractions), via a linear accelerator to the site of the proximal femoral lesion after the surgical incision had properly healed.

**Figure 1:**
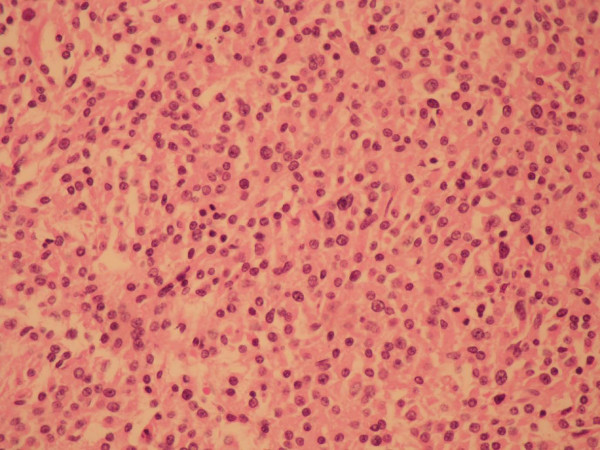
**Light microscopy image of formalin-fixed tissue demonstrating mature and atypical plasma cells with eccentric hyperchromatic nuclei**.

Three months after the completion of radiotherapy, the patient reported significant back pain. Whole bone scintigraphy showed an increased uptake over the thoracic spine, and a magnetic resonance imaging (MRI) study of the spine revealed multiple areas of bony destruction involving T2-T4. A laminectomy of the thoracic spine with resection of the spinal tumor was done performed (3.5 months after completion of radiotherapy). The pathologic report was consistent with a plasma cell tumor and non-secretory multiple myeloma (Fig. 1). Post-surgery, the subject underwent radiotherapy to the thoracic spine (4500 cGy in 25 fractions), followed by oral chemotherapy with mephalan (12 mg/day) and prednisolone (100 mg/day), 4 days/month.

**Figure 2:**
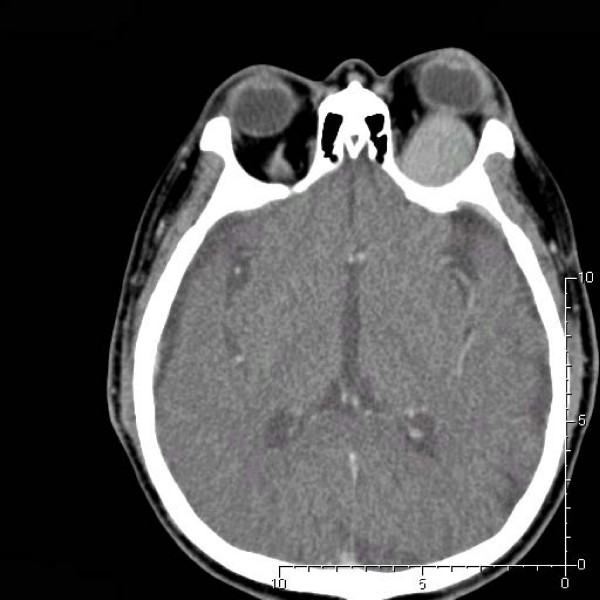
**Axial view of the orbital computerized tomographic scan showing the tumor encasing the optic nerve. Prominent bony destruction could not be observed**.

**Figure 3:**
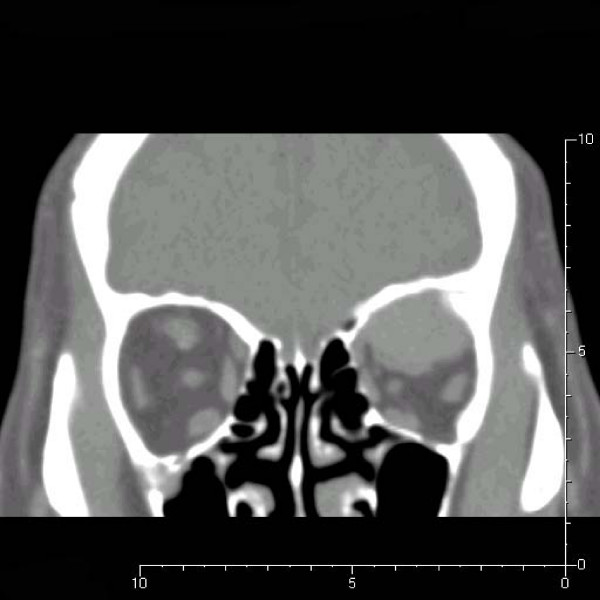
**Coronal view of the orbital computerized tomographic scan showing left intra-orbital mass lesion, occupying the upper-outer quadrant and compressing the superior rectus muscle**.

Approximately 2.5 years ago, the patient developed progressive proptosis and a limitation of gaze. An ophthalmologic examination at that time revealed a visual acuity of 20/40 in the right eye and 20/200 in the left eye. The left eye was noted to protrude 5 mm more anteriorly than the right eye. A computerized tomographic (CT) scan of the orbit revealed a left intraorbital mass lesion occupying the upper-outer quadrant, compressing the superior rectal muscle and encasing the optic nerve, but without prominent bony destruction (Fig. 2, 3).

In order to expeditiously release the nerve-compression, a left frontal skull craniotomy to the midline was performed, prior to an epidural retraction of the left frontal lobe in a step-by-step fashion until the orifice of the optic canal was reached. Some tumor-like tissue was identified on the orbital roof. After removing the tumor-like tissue, unroofing of the orbit was performed. Histopathological examination of the tissue revealed bone and soft tissue with infiltrating plasma cells containing round, eccentric, hyperchromatic nuclei, consistent with a diagnosis of plasma cell tumor. At the time of surgery, the tumor was found to have invaded the orbital cavity, encased the optic nerve, and also infiltrated superiorly compressing the levator and superior rectus muscles. Further unroofing of the orbit was done to excise residual non-visible tumor ensuring total removal. Two titanium mesh patches were used to control orbital pressure. After surgery, the subject's left eye exhibited no significant exophlathesis and he reported a slightly blurred vision.

At follow-up one month after surgery, examination revealed markedly decreased proptosis of the eye and the subject was referred to the radiation oncology department for additional treatment The patient underwent radiotherapy to the primary orbit tumor bed, with a dose of 3960 cGy in 22 fractions. As the patient experienced did not tolerate the prior medical treatment, Zometa^® ^(zoledronic acid; Novartis), 4 mg/month was administered during the course of radiation therapy to prevent disease progression.

The subject was followed every 2 to 4 month with examinations and visual acuity checks, and bone scans every 6 months. At follow-up examination 32 months after frontal skull craniotomy followed by moderate-dose radiotherapy, his visual acuity was 20/50 in the left eye and he had no other complaints. CT scan at that time showed post-surgery changes in the retro-orbital area and no abnormal mass tissue was noticed.

## Discussion

Care should be taken in the diagnosis of orbital multiple myeloma, as it has been demonstrated that orbital plasmacytoma may mimic other orbital tumors such as meningioma, melanoma, and orbital carcinoma under angiography [6]. Optic nerve sheath meningiomas (ONSMs) account for 1-2% of all meningiomas. The most frequent presenting symptom of ONSM is painless loss of visual acuity. The ONSM surrounds the optical nerve and the caliber of optical nerve is attenuated within the surrounding tumor. This is in contrast to optical nerve gliomas, where the optical nerve itself is expanded [7]. Mitoses, architectural disruption, calcification, and MIB-1 staining are also described. The distribution of ocular melanoma are 80% in the choroid, 10-15% in the ciliary body, and <10% in the iris. The pathologic findings include spindle cells (30%), epithelioid cells (5%), and mixed cells (65%) (contains spindle and epithelioid cells) [8]. Vascular lesions account for 5%-20% of orbital masses, and hemangioma and lymphangioma are the most common vascular lesions in the orbit. The vascular features and the flow voids on MR images of hemangioma distinguish hemangioma from these other lesions. Hemangiomas are vascular and multilobular at gross examination. Histologically, the tumor growth appears infiltrative and may involve adjacent orbital structures. In the early proliferative phase, the lesion is composed of densely packed, plump, hyperplastic endothelial cells that form clusters or lobules [9].

In the current case study examined a subject with orbital multiple myeloma. Malignant carcinoma was excluded because it is usually accompanied by prominent bony destruction and is characterized by an extended area of invasion. Neither the physical exam or laboratory findings suggested the presence of malignant lymphoma. The occurrence of an inflammatory pseudotumor was also unlikely, as no episodes of erythema involving the eyelid had occurred in this patient. The clinical findings indicated a rare case, as plasma cell tumors with orbital involvement are not often reported, even among patients with known multiple myeloma. Reports suggest that orbital involvement in patients with multiple myeloma may be a first sign of insufficient treatment or the first sign of systemic disease [10,11] In addition, fine needle aspiration of has been shown to play a role in the diagnosis when extramedullary involvement is suspected [11].

Because multiple myeloma is a systemic, disseminated disease, chemotherapy is the preferred treatment modality. Local, aggressive treatments such as surgery are generally not used; however, in the subject examined herein, proptosis and limitation of gaze occurred over a short time period and chemotherapy was not suitable for local control, especially in the orbit area. External beam radiation therapy can achieve superior local therapeutic results when used at a relatively high dose of 5000-6000 cGy [12]. However, the dose of radiation, its method of delivery, and possible irradiation of surrounding normal tissues may largely depend on the tumor mass present at the time of treatment. If radiotherapy is the only available choice because the tumor is located in the intra-orbital and retrobulbar regions, protecting the retinas and lenses of both eyes is technically challenging.

In our case, the subject survived for more than 84 months since diagnosis, which is nearly twice the mean survival time [13]. Alexanian [14] and Cherng et al. [15] revealed that several clinical criteria influence a patient's duration of remission and survival time: a hemoglobin count of <8.5 g/100 ml, elevation of the ionized calcium level, an IgG peak of >7 g/100 ml, and hypoalbuminemia were all associated with shorter survival. Our subject never exhibited anemia, hypercalcemia, or renal insufficiency, and his performance status was excellent. Therefore, we decided to employ an unusual therapy, i.e., surgical removal, that did not induce prominent complications. We completely removed the gross optic tumor and applied radiotherapy post-surgery using a moderate radiation dose to better control residual microscopic tumor development. In a recent report, Gönül et al. [16] used right orbitozygomatic craniotomy to successfully remove a mass lesion in a 60-year-old subject diagnosed with orbital multiple myeloma.

The surgical approach that we undertook was distinct from that followed by most surgeons [17,18]. We chose a frontal skull craniotomy with an epidural approach to the orbital space because the intraorbital mass lesion occupied the upper-outer quadrant, compressing the superior rectus muscle, and encasing the optic nerve. Although we realized that the 2^nd^, 3^rd^, 4^th^, and 6^th ^cranial nerves could be injured by an orbital approach, the surgical procedure allowed removal of the majority of the tumor, which substantially increased the control rate of the adjuvant radiotherapy.

Numerous reports describe invasion of the orbit by multiple myeloma, as demonstrated by CT scan [5,18-20]. Most of these cases had large soft tissue masses arising from within the bone, causing bony expansion and destruction that could not be ameliorated by surgery. In addition, the patients exhibited a number of poor prognostic factors, such as high serum or urine paraprotein, low hemoglobin, or multiple bony lesions, and thus palliative radiotherapy was chosen to relieve nerve compression. In some reports, a palliative radiotherapy dose (i.e., a total dose <3000 cGy) was chosen[17,21]; however, the dose was intended for palliative treatment and the radiation field using this dose was designed to encompass the whole bone, if possible. The daily dose should exceed 200 cGy to achieve a better radiation effect. If we had initially used a total dose <3000 cGy, the optic tumor may have redeveloped after a period of time. Also, a fractional dose >200 cGy per day would injure optic structures, such as the optic nerve, lens, or retina. In our subject, although myeloma was diagnosed in the left femur and thoracic spine, symptom-free survival has extended to 32 months after completion of the radiotherapy, which is significantly longer than the reported median survival [13].

Although the effectiveness and applicability of this approach remains to be determined though the evaluation of its application in additional cases of orbital multiple myeloma, this case report demonstrates that accurate and early detection, currently facilitated by magnetic resonance imaging [22], combined with local surgical treatment and appropriate radio/chemotherapy, can be applied to effectively extend an orbital multiple myeloma patient's life.

## Consent

Written informed consent was obtained from the patient for publication of this case report and accompanying images. A copy of the written consent is available for review by the Editor-in-Chief of this journal.

## Competing interests

The authors declare that they have no competing interests.

## Authors' contributions

We declare that all the listed authors have participated actively in the study and all meet the requirements of the authorship. HLK carried out the radiotherapy, and drafted the manuscript. CLC participated in its design and coordination of the study. KHC conceived of the study, and helped to draft the manuscript. All authors read and approved the final manuscript.
